# What are the risk factors for extraintestinal manifestations in inflammatory bowel diseases?

**DOI:** 10.1097/MD.0000000000033031

**Published:** 2023-03-03

**Authors:** Min-Kyung Yeo, Jae Ho Park, Sun Hyung Kang, Hee Seok Moon, Jae Kyu Sung, Hyun Yong Jeong, Ju Seok Kim

**Affiliations:** a Department of Pathology, Chungnam National University School of Medicine, Daejeon, Korea; b Departments of Internal Medicine, Chungnam National University School of Medicine, Daejeon, Korea.

**Keywords:** extraintestinal manifestations, inflammatory bowel disease, prevalence, risk factor

## Abstract

Extraintestinal manifestations (EIMs) are common in patients with inflammatory bowel disease (IBD); however, studies surrounding EIMs are lacking, particularly in Asia. This study aimed to identify risk factors by analyzing the characteristics of patients with EIMs. From January 2010 to December 2020, the medical records of 531 patients diagnosed with IBD (133 with Crohn disease [CD] and 398 with ulcerative colitis [UC]) were reviewed. The patients’ baseline characteristics and risk factors were analyzed by dividing them into 2 groups according to EIMs presence. The prevalence of EIMs in all patients with IBD was 12.4% (n = 66), of which CD and UC prevalences were 19.5% (n = 26) and 10.1% (n = 40), respectively. The articular (7.9%, n = 42), cutaneous (3.6%, n = 19), ocular (1.5%, n = 8), and hepatobiliary types (0.8%, n = 4) of EIMs were observed. Two or more EIMs occurred in only 1.2% of all IBD patients (n = 6). Multivariate analysis revealed that the risk factors for the occurrence of EIMs were a follow-up period ≥ 10 years (odds ratio, 2.106; 95% confidence interval, 1.187–3.973; *P* = .021) and treatment with biologics (odds ratio, 1.963; 95% confidence interval, 1.070–3.272; *P* = .037). The EIMs prevalence in patients with IBD was 12.4%, and the particular type was the most common, with EIMs occurring more frequently in patients with CD than in those with UC. Patients who have been treated for IBD for more than 10 years or who are using biologics should be carefully monitored as they are at high risk for EIMs.

## 1. Introduction

The number of patients with inflammatory bowel disease (IBD) in Asia is increasing, but studies on the pathophysiology, prognosis, and complications of IBD are still lacking in the Asian population compared with the Western population.^[1,2]^ IBD is a multifactorial disease that causes chronic inflammation of the bowel; however, its pathogenesis remains unknown. IBD is classified into Crohn disease (CD) and ulcerative colitis (UC), and there are differences in their clinical courses and therapeutic agents.^[3,4]^

IBD is a systemic disease that is not limited to the bowel and causes various extraintestinal manifestations (EIMs).^[5]^ According to several studies, it is known that 6% to 47% of patients with IBD develop EIMs, and there are differences in its incidence according to race and geographic location.^[6,7]^ EIMs mainly involve the joints, skin, eyes, hepatobiliary organs, and with joint involvement being the most common. Some patients also show the involvement of more than 2 organs.^[8]^ Although the pathogenesis of EIMs has not yet been elucidated, in the case of a long duration of IBD, perianal involvement in CD, extensive UC, and smoking are known to be risk factors, but data on Asian patients are lacking.^[9–11]^

EIMs are major complications that adversely affect the quality of life of IBD patients, causing disease aggravation or alteration of treatment agents. Studies have shown that patients with EIMs use more biologics and have a higher risk of undergoing surgery than those without EIMs.^[12]^

The purpose of this study was to determine the prevalence of EIMs in patients with IBD, to identify risk factors for EIMs by dividing patients into 2 groups according to the presence of EIMs, and to compare and analyze the characteristics of the patients.

## 2. Methods

### 2.1. Study design and patient selection

From January 2010 to December 2020, medical records of patients aged > 18 years who were diagnosed with IBD and treated at Chungnam National University School of Medicine (Daejeon, Korea) were retrospectively analyzed. Patients who refused treatment were transferred to another hospital or who were not followed up continuously for more than 1 year were excluded from the study.

The patients were classified into CD and UC groups, and baseline characteristics such as sex, age at diagnosis, follow-up period, smoking history, family history of IBD, treatment with biologics, and surgery during the follow-up period were compared. Based on the occurrence of EIMs, the patients were divided into 2 groups to analyze the prevalence and risk factors of EIMs. This study was approved by the Institutional Review Board of Chungnam National University Hospital (2019-06-034).

### 2.2. Definition

IBD diagnosis was made by an expert gastroenterologist by synthesizing various findings, such as conventional clinical, endoscopic, radiologic, and histopathologic results. CD was defined as disease location (L1, ileal; L2, colonic; L3, ileocolonic; L4, and upper gastrointestinal tract) and disease behavior (B1, nonstricturing, nonpenetrating; B2, stricturing; B3, and penetrating) according to the Montreal classification. UC is classified into proctitis (E1), left-sided colitis (E2), and extended colitis, including pancolitis (E3), and according to the degree of disease extent.^[13]^

EIMs were diagnosed and treated by a specialist according to their subtypes. Depending on the organ involved, EIMs were defined as articular (peripheral arthritis, ankylosing spondylitis, and sacroiliitis), cutaneous (erythema nodosum, pyoderma gangrenosum, and others), ocular (uveitis and episcleritis), or hepatobiliary (primary sclerosing cholangitis). Patients with 2 or more EIMs were classified according to the number of EIMs.

### 2.3. Statistical analysis

Categorical variables were expressed as proportions and percentages and analyzed using the chi-square test or Fisher exact test, and continuous variables were expressed as means and standard deviations. The prevalence of EIMs in all IBD patients and in the CD and UC groups were compared according to EIM subtypes. According to the occurrence of EIMs, the characteristics were analyzed by dividing the patients into 2 groups, and multivariate analysis was performed by adjusting for significant factors, such as sex, follow-up period, the extent of UC, and treatment with biologics.

Data were expressed as odds ratios (OR) and confidence interval (CI), *P* values were 2-sided, and a *P* < .05 was considered significant. All statistical analyses were performed using SPSS version 19.0 (SPSS Inc., Chicago, IL).

## 3. Results

### 3.1. Patient characteristics

A total of 531 patients were enrolled in this study, of whom 25.0% (n = 133) had CD, and 75.0% (n = 398) had UC. There were 67.4% (n = 358) men among all patients, the mean age at diagnosis of IBD was 40.3 (6.4) years, and the mean follow-up period was 8.5 (3.2) years. The analysis of all IBD patients divided into CD and UC groups is shown in Table 1. In patients with CD, 77.4% (n = 103) of the patients were men, significantly higher than the percentage of men with UC, 64.1% (n = 255) (*P* = .007). The mean age at diagnosis was 39.8 (9.3) years for CD and 41.1 (8.7) years for UC, and there was no significant difference between the 2 groups (*P* = .315). The mean follow-up period was also not significant (*P* = .448). Subgroup analysis revealed that L3 (45.9%) and B1 (70.0%) were more common in CD patients than in UC patients, whereas E1 (41.2%) was more common in UC patients than in CD patients. As a limitation of the retrospective study, there were missing data for smoking history (n = 15) and family history of IBD (n = 31). Smoking history tended to be higher in UC patients (50.9%) than in CD patients (38.2%), but the difference was not statistically significant (*P* = .063), and a family history of IBD showed a similar trend (*P* = .146). The rate of treatment with biologics was significantly higher in CD patients (30.1%) than in UC patients (13.1%) (*P* < .001), and there was no difference between the 2 groups for patients who underwent surgery during the follow-up period (CD, 9.8%; UC, 5.3%; *P* = .103).

**Table 1:** Total patient baseline characteristics.

Characteristics, No. (%)	CD	UC	*P* value
	n = 133	n = 398	
Gender (male)	103 (77.4)	255 (64.1)	.007
Age at diagnosis (SD), yr	39.8 (9.3)	41.1 (8.7)	.315
Follow-up period (SD), yr	8.2 (4.1)	8.7 (3.5)	.448
Location of CD			
L1	27 (20.3)		
L2	42 (31.6)		
L3	61 (45.9)		
Concomitant L4	3 (2.2)		
Behavior of CD			
B1	93 (70.0)		
B2	27 (20.3)		
B3	13 (9.7)		
Extent of UC			
E1		164 (41.2)	
E2		116 (29.1)	
E3		118 (29.7)	
*Smoking history			
Yes	50 (38.2)	190 (50.9)	.063
*Family history of IBD			
Yes	8 (6.2)	43 (11.5)	.146
Biologics treatment			
Yes	40 (30.1)	52 (13.1)	< .001
Surgery			
Yes	13 (9.8)	21 (5.3)	.103

CD = Crohn disease, IBD = inflammatory bowel disease, SD = standard deviation, UC = ulcerative colitis.

* Missing value (Smoking history: n = 15, Family history of IBD: n = 31).

### 3.2. Prevalence of extraintestinal manifestations

**Figure 1.**
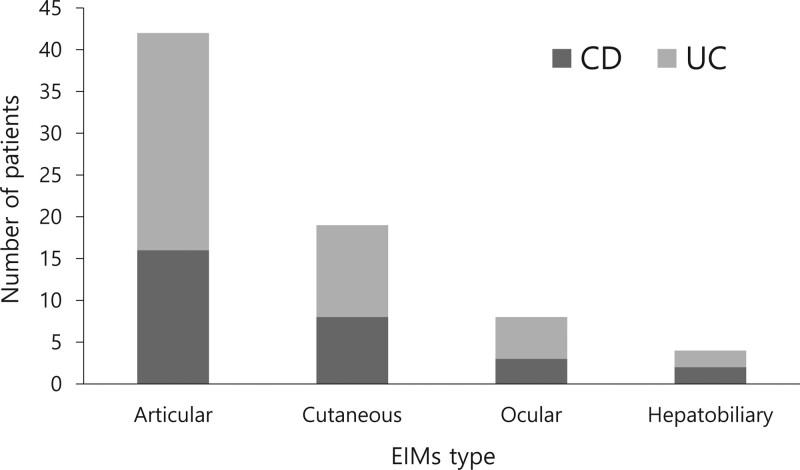
Prevalence of EIMs according to inflammatory bowel disease type. EIMs = extraintestinal manifestations.

The prevalence of EIMs according to the IBD type is shown in Figure 1. EIMs occurred in 12.4% (n = 66) of all IBD patients, and among them, 2 or more EIMs were observed in 6 patients, resulting in a total of 73 (13.7%) patients with EIMs (Table 2). When classified by EIM subtype, articular was the most common type of EIM observed (7.9%, n = 42), followed by cutaneous (3.6%, n = 19), ocular (1.5%, n = 8), and hepatobiliary (0.8%, n = 4).

**Table 2:** Prevalence of extraintestinal manifestations of inflammatory bowel disease patients.

Characteristics, No. (%)	All patients	CD	UC	*P* value
	n = 531	n = 133	n = 398	
All EIMs	73 (13.7)	29 (21.8)	44 (11.1)	.003
Articular	42 (7.9)	16 (12.0)	26 (6.5)	.064
Peripheral arthritis	31 (5.8)	12 (9.0)	19 (4.7)	
Ankylosing spondylitis	7 (1.3)	3 (2.3)	4 (1.0)	
Sacroiliitis	4 (0.8)	1 (0.7)	3 (0.8)	
Cutaneous	19 (3.6)	8 (6.0)	11 (2.8)	.139
Erythema nodosum	10 (1.9)	4 (3.0)	6 (1.5)	
Pyoderma gangrenosum	6 (1.1)	2 (1.5)	4 (1.0)	
Others	3 (0.6)	2 (1.5)	1 (0.3)	
Ocular	8 (1.5)	3 (2.3)	5 (1.3)	.683
Uveitis	5 (1.0)	2 (1.5)	3 (0.8)	
Episcleritis	3 (0.5)	1 (0.8)	2 (0.5)	
Hepatobiliary	4 (0.8)	2 (1.5)	2 (0.5)	.564
Primary sclerosing cholangitis	4 (0.8)	2 (1.5)	2 (0.5)	
Number of EIMs				.103
1	60 (11.3)	23 (17.3)	37 (9.3)	
2	5 (1.0)	3 (0.2)	2 (0.5)	
3	1 (0.2)	0 (0.0)	1 (0.3)	

CD = Crohn disease, EIMs = extraintestinal manifestations, UC = ulcerative colitis.

The prevalence of all EIMs in patients with CD was 21.8% (n = 29), which was significantly higher than that in patients with UC at 11.1% (n = 44) (*P* = .003). The articular (*P* = .064), cutaneous (*P* = .139), ocular (*P* = .683), and hepatobiliary (*P* = .564) types were not significantly different between the CD and UC groups. Of the 66 patients with IBD for whom EIMs were found, 6 patients had multiple EIMs, of which 5 patients developed 2 EIMs, and 1 patient had 3 EIMs.

### 3.3. Risk factors for extraintestinal manifestations

Baseline characteristics were analyzed by dividing the patients into 2 groups according to the occurrence of EIMs (Table 3). There were significantly more women with EIMs (47.0%) than without EIMs (30.5%) (*P* = .012). However, there was no difference in the age at IBD diagnosis (*P* = .470). There were more patients with follow-ups ≥ 10 years in the EIM group than in the nonEIM group (68.2% vs 38.3%), with a statistically significant difference (*P* < .001). In patients with CD, location (*P* = .278) and behavior (*P* = .423) did not differ according to the occurrence of EIMs, but in the case of extensive disease among patients with UC, the occurrence of EIMs was significantly higher than that in the case of localized disease (*P* = .031). Smoking history (*P* = .213) and family history of IBD (*P* = .063) did not differ significantly according to the occurrence of EIMs. In the case of treatment with biologics, the incidence of EIMs was significantly higher than that in the case of treatment without biologics (31.8% vs 15.3%, *P* = .002), and there was a tendency for the incidence of EIMs in the surgery group to be high, but the difference was not statistically significant (12.1% vs 5.6%, *P* = .078).

**Table 3:** Risk factors for extraintestinal manifestations in inflammatory bowel disease patients.

Characteristics, No. (%)	With EIMs	Without EIMs	*P* value
	n = 66	n = 465	
Gender			.012
Male	35 (53.0)	323 (69.5)	
Female	31 (47.0)	142 (30.5)	
Age at diagnosis, yr			.470
< 30	25 (37.9)	202 (43.4)	
≥ 30	41 (62.1)	263 (56.6)	
Follow-up period, yr			
< 10	21 (31.8)	287 (61.7)	< .001
≥ 10	45 (68.2)	178 (38.3)	
Location of CD			.278
L1	4 (6.1)	23 (5.0)	
L2	10 (15.2)	32 (6.9)	
L3	12 (18.2)	49 (10.5)	
Concomitant L4	0 (0.0)	3 (0.6)	
Behavior of CD			.423
B1	16 (24.2)	77 (16.6)	
B2	6 (9.1)	21 (4.5)	
B3	4 (6.1)	9 (2.0)	
Extent of UC			.031
E1	13 (19.7)	151 (32.5)	
E2	11 (16.7)	105 (22.6)	
E3	16 (24.2)	102 (21.9)	
*Smoking history			.213
Yes	35 (53.0)	205 (44.1)	
*Family history of IBD			.063
Yes	11 (16.7)	40 (8.6)	
Biologics treatment			.002
Yes	21 (31.8)	71 (15.3)	
Surgery			.078
Yes	8 (12.1)	26 (5.6)	

CD = Crohn disease, EIMs = extraintestinal manifestations, IBD = inflammatory bowel disease, UC = ulcerative colitis.

* Missing value (Smoking history: n = 15, Family history of IBD: n = 31).

To identify risk factors for the occurrence of EIMs, multivariate analysis was performed by adjusting for sex, follow-up period, extent of UC, and treatment with biologics, which were obtained from the univariate analysis (Table 4). As a result, a follow-up period of ≥ 10 years (OR, 2.106; 95% CI, 1.187–3.973; *P* = .021) and history of treatment with biologics (OR, 1.963; 95% CI, 1.070–3.272; *P* = .037) were independent risk factors for EIMs in patients with IBD. However, female sex (OR, 1.626; 95% CI, 0.930–2.839; *P* = .092) and extensive UC (OR, 1.326; 95% CI, 0.810–1.733; *P* = .135) were not statistically significant.

**Table 4:** Multivariate analysis of factors associated with extraintestinal manifestations.

Variables	OR (95% CI)	*P* value*
Gender		.092
Male	References (1.0)	
Female	1.626 (0.930–2.839)	
Follow-up period, yr		.021
< 10	References (1.0)	
≥ 10	2.106 (1.187-3.973)	
Extent of UC		.135
Localized	References (1.0)	
Extensive	1.326 (0.810–1.733)	
Biologics treatment		.037
No	References (1.0)	
Yes	1.963 (1.070–3.272)	

CI = confidence interval, OR = odds ratio, UC = ulcerative colitis.

* Adjusted for gender, follow-up period, extent of UC and biologics treatment.

## 4. Discussion

In this study, the overall incidence of EIMs in patients with IBD was 12.4%. In several previous studies, the incidence of EIMs was reported to be about 6% to 40%, and differences were observed according to race, geographic location, and study design.^[1,14]^ Existing studies have mainly focused on Western patients, a recent study on Asian patients showed a similar result to the present study with an EIM incidence rate of 11.3%.^[15]^ The difference in the incidence of EIMs in patients with IBD is thought to have influenced the definition of EIMs and the differences in inclusion criteria.

In general, it has been reported that multiple concomitant EIMs occur in approximately 0.3% to 4.5% of patients with IBD.^[16]^ In this study, multiple EIMs were observed in 1.1% of all patients with IBD with 2 EIMs being the most commonly seen. The risk of multiple EIMs is reported to be higher in patients with CD than in those with UC.^[17]^ In this study, the incidence of multiple EIMs in patients with CD was higher, but the difference was not statistically significant (0.8% vs 2.3%, *P* = .103). In previous studies, it has been shown that the overall incidence of EIMs is higher in patients with CD than in patients with UC. In this study, the prevalence of EIMs in patients with CD was 21.8%, which was significantly higher than that in patients with UC (11.1%, *P* = .003).^[10,17]^

When classified by the EIM subtype, the articular subtype was most common (7.9%), followed by cutaneous (3.6%), ocular (1.5%), and hepatobiliary (0.8%) subtypes. In other studies, consistent with the findings of the present study, articular-type EIMs were observed most often; among them, peripheral arthritis is known to be the most common.^[18–20]^ In a study targeting Western patients, the incidence of EIMs was reported to be higher. In a study analyzing the incidence of EIM subtypes in 790 Korean patients with IBD, the incidence in the peripheral joint was 7.8%, that in the axial joint was 1.1%, that in the skin was 1.5%, that in the hepatobiliary organs was 0.9%, and that in the eye was 0.8%.^[6,15]^ It is thought that there is a difference in incidence according to race, and a different study design may have an effect on each individual study. Therefore, a large-scale study that directly compares the incidence of EIMs in patients with IBD in the East and in the West is needed.

Female sex, active disease, perianal and upper involvement of CD, IBD diagnosis at a young age, and family history are known risk factors for EIMs. However, there are differences between studies, so the factors remain controversial. In particular, there are few studies analyzing the risk factors of EIMs in Asian patients with IBD.^[9,18,21]^ In a study analyzing EIMs in 1764 Asian patients with IBD, the incidence of EIMs in both female patients with CD (OR, 2.02; 95% CI, 1.15–3.55) and female patients with UC (OR, 2.57; 95% CI, 1.52–4.34) was found to be significantly higher than those in male patients.^[15]^ In this study, the incidence of EIMs in female patients with IBD tended to be high, but the difference was not statistically significant (*P* = .092). Studies have reported a higher incidence of EIMs in patients with CD than in those with UC.^[22]^ In this study, the prevalence of EIMs in patients with CD was 21.8% (n = 29), which was significantly higher than that in patients with UC at 11.1% (n = 44) (*P* = .003). In addition, a follow-up period of ≥ 10 years (OR, 2.106; 95% CI, 1.187–3.973; *P* = .021) and history of treatment with biologics (OR, 1.963; 95% CI, 1.070–3.272; *P* = .037) were significant risk factors for EIMs in patients with IBD. If IBD activity is high, the risk of EIMs also increases, and it is thought that lowering disease activity through appropriate treatment will help prevent EIMs.

There were several limitations to this study. First, it is difficult to generalize the research results of a retrospective study that analyzed existing medical records. However, a relatively large number of patients with IBD were enrolled, as the study location is the only tertiary medical center in the region, a variety of patients visit; therefore, this limitation is not expected to be significant. Second, the risk factors for the development of EIMs in patients with IBD were identified, but the mechanism of the prevalence of EIMs has not been elucidated. Several hypotheses attempt to explain the occurrence of EIMs, but they are still controversial, and further studies are needed.^[23]^ The strength of this study is that it analyzed the risk factors for EIMs in Asian patients with IBD, a population that has not been studied extensively in the past.

In conclusion, the overall incidence of EIMs in patients with IBD was 12.4%, and a follow-up period of ≥ 10 years and a history of treatment with biologics were independent risk factors related to the occurrence of EIMs. Patients with IBD with these risk factors should be carefully monitored for the occurrence of EIMs and treated appropriately according to the type and activity of EIM upon occurrence.

## Author contributions

**Conceptualization:** Min-Kyung Yeo, Jae Kyu Sung, Hyun Yong Jeong, Ju Seok Kim.

**Funding acquisition:** Hee Seok Moon, Hyun Yong Jeong, Ju Seok Kim.

**Investigation:** Min-Kyung Yeo.

**Methodology:** Min-Kyung Yeo, Jae Ho Park, Hyun Yong Jeong, Ju Seok Kim.

**Project administration:** Sun Hyung Kang, Hee Seok Moon, Ju Seok Kim.

**Resources:** Jae Ho Park.

**Software:** Sun Hyung Kang.

**Supervision:** Jae Kyu Sung, Hyun Yong Jeong, Ju Seok Kim.

**Visualization:** Sun Hyung Kang.

**Validation:** Jae Kyu Sung.

**Writing – original draft:** Min-Kyung Yeo.

**Writing – review & editing:** Hee Seok Moon, Ju Seok Kim.
